# Supplementary material for: Causal interplay between lactose intolerance and gut microbiota: a combined bidirectional Mendelian randomization and in vivo validation study
Source: Front Nutr. 2026 Jun 1;13:1803337. doi: 10.3389/fnut.2026.1803337 (PMC13265576; doi:10.3389/fnut.2026.1803337)
Supplement: Supplementary file 1 [file Data_Sheet_1.zip › supplementary materials/Forward/leave one out cross validation/ebi-a-GCST90027720.finngen_R12_E4_LACTONAS.pdf]

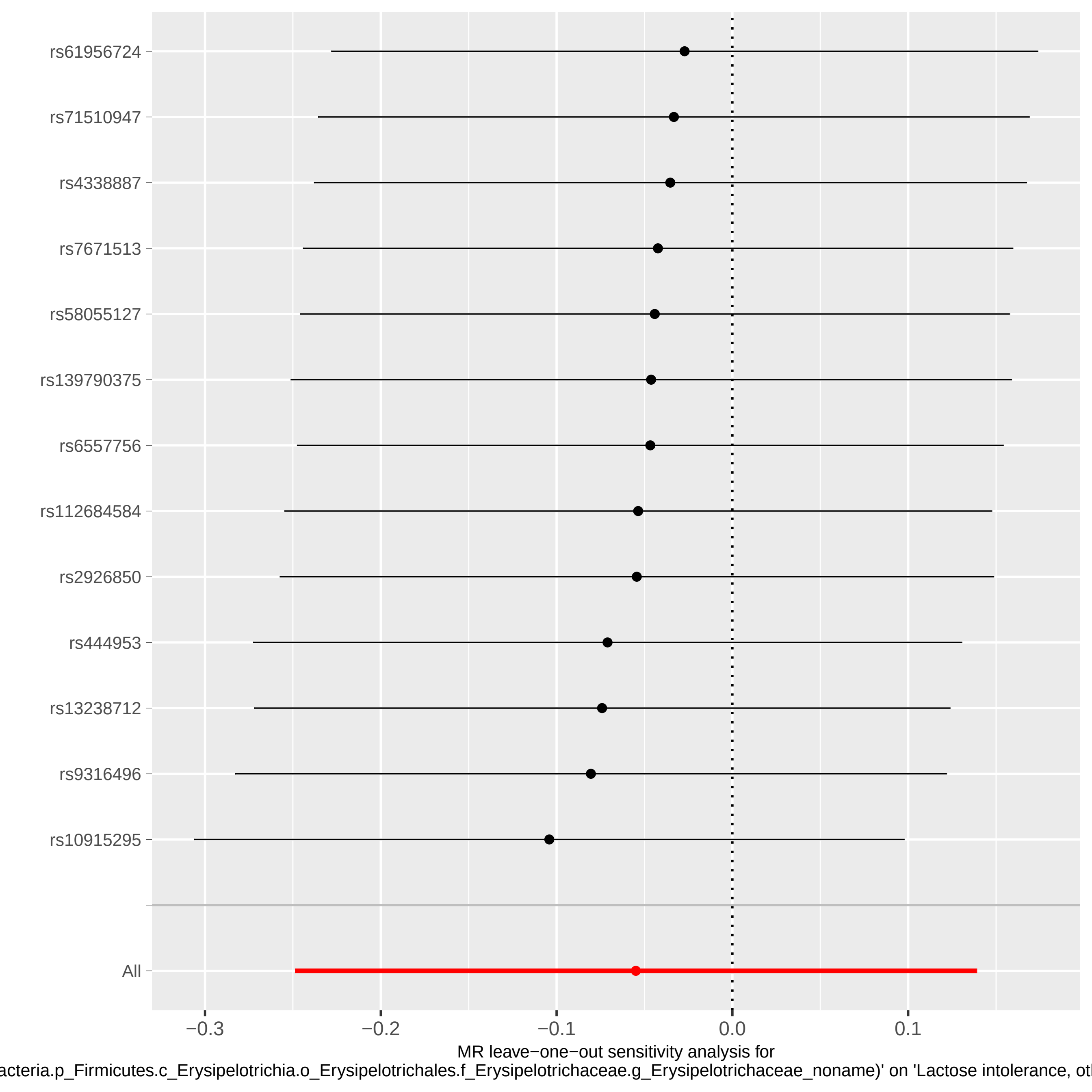
rs61956724
rs71510947
rs4338887
rs7671513
rs58055127
rs139790375
rs6557756
rs112684584
rs2926850
rs444953
rs13238712
rs9316496
rs10915295
All
−0.3
−0.2
−0.1
0.0
0.1
MR leave−one−out sensitivity analysis for
acteria.p_Firmicutes.c_Erysipelotrichia.o_Erysipelotrichales.f_Erysipelotrichaceae.g_Erysipelotrichaceae_noname)' on 'Lactose intolerance, ot
